# Magnetic resonance imaging features of canine intradural/extramedullary intervertebral disc extrusion in seven cases

**DOI:** 10.3389/fvets.2022.1003042

**Published:** 2022-09-14

**Authors:** David Casado, Ricardo Fernandes, Filipa Lourinho, Rita Gonçalves, Robert Clark, Francesca Violini, Inés Carrera

**Affiliations:** ^1^Willows Veterinary Centre and Referral Service, Part of Linnaeus Veterinary Limited, Solihull, United Kingdom; ^2^Paragon Veterinary Referrals, Part of Linnaeus Veterinary Limited, Wakefield, United Kingdom; ^3^Southern Counties Veterinary Specialists, Ringwood, United Kingdom; ^4^Department of Veterinary Clinical Sciences, University of Liverpool, Liverpool, United Kingdom; ^5^Vet Oracle, Norfolk, United Kingdom

**Keywords:** MRI, dog, Y sign, disc extrusion, intradural, extramedullary

## Abstract

Intervertebral disc disease, including intervertebral disc extrusions and protrusions, is the most common spinal cord disorder in dogs. Atypical and uncommon intervertebral disc herniations include intradural/intramedullary disc extrusion, intervertebral foraminal disc extrusion and intervertebral disc herniation (Schmorl's node). Intradural/extramedullary disc extrusion is the least common type of intervertebral disc herniation in veterinary medicine, characterized by extruded disc material within the intradural space. To date, only one study has been published in veterinary medicine reporting intradural/extramedullary disc extrusions. In this study, low field MRI was used, and the authors could not find any MRI features to diagnose with confidence an intradural/extramedullary disc location of the extruded disc material. The aim of this study was to describe the high field (1.5T) MRI characteristics of surgically confirmed intradural/extramedullary disc extrusions. This is a retrospective, multicentric and descriptive study. Inclusion criteria was surgical confirmation of intradural/extramedullary disc extrusion by durotomy and complete MRI study of the spine. Seven cases were included. Images were reviewed by a radiology resident and a certified radiologist, with emphasis on the following signs: “Golf-tee sign” (widening of the subarachnoid space cranial and caudal to the lesion), “Beak sign” (pointed and sharp compressive lesion) and “Y sign” (division of the dura and arachnoid layers). MRI showed a “Y sign” in all the cases (7/7) seen from the T2-weighted sagittal views, while “Golf-tee sign” was not recognized in any of the cases (0/7). Additionally, “beak sign” was present in half of the cases (4/7). “Y sign” maybe a reliable MRI feature for identifying intradural/medullary disc extrusions from the MRI study. As the arachnoid is peeled from the dura by the disc herniation there is a splitting of the arachnoid mater and the ventral dura. The intradural disc material will be surrounded by CSF signal intensity margin, giving the appearance of a Y, which can be identified from the T2-weighted sagittal images.

## Introduction

The most common spinal cord disease in dogs is intervertebral disc (IVD) disease accounting for 2.3–3.7% of all the hospitalizations to small animal veterinary hospitals (1, 2). The term intervertebral disc herniation (IVDH) describes any form of IVD disease involving a displacement of part of the IVD into the vertebral canal. IVDH is commonly associated with intervertebral disc degeneration (3, 4) and has been traditionally classified into acute IVD extrusion (Hansen Type I) and chronic IVD protrusion (Hansen Type II), which both may imply the presence of extradural material causing spinal cord compression (5). IVDH with extruded material into the extradural space is the most common cause of spinal cord injury reported in dogs with intervertebral disc disease (88%) (6, 7), and chondrodystrophic breed dogs are particularly predisposed. Standard and Miniature Dachshunds are overrepresented due to a genetic malformation associated with disc calcification (2, 6, 8). Specifically, a FGF4 retrogene has been identified to be responsible for the susceptibility of chondrodystrophic breed dogs to Hansen's type I intervertebral disc disease (9).

Diagnostic imaging plays a crucial role on the diagnosis of IVDH. The exquisite soft tissue resolution of magnetic resonance imaging (MRI) allows the characterization of the exact location of the herniated disc material, as well as the characterization of the materials' nature (9). The already wide spread use of this imaging modality in veterinary medicine has made possible the recognition of more types of IVDH, in addition to the already well-documented Hansen type I and II, which include acute non-compressive nucleus pulposus extrusion, hydrated nucleus pulposus extrusion (7, 10), intradural/intramedullary intervertebral disc extrusion (11), intervertebral foraminal disc extrusion (12, 13) and intravertebral disc herniation (Schmorl's node) (14). Furthermore, there is another unusual location of herniated disc material, which is intradural/extramedullary (11, 15, 16). Extruded nucleus pulposus within the intradural space causing spinal cord compression is uncommon in dogs with just a total of 10 cases reported in the literature (11, 17–19).

Despite recent advances in diagnostic imaging techniques, the identification of intradural/extramedullary disc extrusions remains challenging, with most of the cases being diagnosed intraoperatively (11, 20–25). In a human review including 122 patients, only 8 were diagnosed preoperatively, denoting the difficulty on detecting intradural/extramedullary herniation pre-operatively (20). It is of practical importance that surgeons recognize preoperatively the type of disc extrusion, so the surgical procedure can be planned in advance in order to reach the best possible outcome. Also, being able to inform the owner of the relative risks, costs and prognosis of an intradural/extramedullary disc extrusion when compared to an extradural intervertebral disc extrusion.

To date, the largest study published in veterinary medicine describing intradural/extramedullary disc extrusion used a combination of myelography, computed tomographic myelography (CTM) and low-field MRI (11). In this study, an intradural/extramedullary lesion compressing the spinal cord at the level of the IVD space, surrounded by accumulated contrast medium (“golf tee sign”) was identified on CTM. However, these findings could not be extrapolated to MRI as the authors could not confirm an intradural/extramedullary disc extrusion in low-field MRI and hence, surgery was needed to obtain a definitive diagnosis in all of the cases.

In human medicine, there are several studies and case series/reports investigating specific MRI signs or features which may help distinguishing intradural/extramedullary from pure extradural disc extrusions (26–30). These are: (A) “Y sign” defined as the division of the dura and arachnoid layers as the arachnoid is peeled off from the dura by the disc extrusion (appearing as “Y”) (26); (B) “Beak sign” described as a sharply marginated and beak-like shape lesion with peripheral ring enhancement after contrast media injection (29); (C) Abrupt loss of continuity of the posterior longitudinal ligament; (D) Peripheral enhancement of the extruded material.

To date, there are no studies investigating MRI features of intradural/extramedullary disc extrusions in dogs using high-field MRI. Therefore, the aim of this study was to describe in detail the MRI features of surgically confirmed intradural/extramedullary disc extrusions in dogs. The hypothesis would be that “Y sign” would be a consistent MRI finding in dogs with intradural/extramedullary disc extrusion, and that “golf tee sign” would not be a reliable MRI finding for the diagnosis of intradural/extramedullary disc extrusions.

## Materials and methods

### Study population

This was a retrospective, multicentre and descriptive study. Medical records from four different referral institutions were reviewed to identify dogs that had an intradural/extramedullary intervertebral disc extrusion on MRI. Ethical approval was granted by the Ethical Review Committee of the University of Nottingham School of Veterinary Medicine and Science. Patient inclusion criteria were: (1) complete MRI study (including at least T2-W sequences with dorsal, transverse and sagittal sequences); and (2) surgical confirmation of intradural/extramedullary disc extrusion by durotomy. Patients were excluded from the study if the MRI study or clinical data was incomplete and if there was no surgical confirmation of intradural/extramedullary disc material. The following medical record data was recorded: age, breed, sex, presenting clinical signs and date of manifestation, initial neurological examination findings, outcome and the date of the MRI study. Outcome was defined as good when the dogs were neurologically normal and ambulatory without assistance.

### Image acquisition and image review

Anesthetic protocols were assessed and tailored for each patient by the corresponding anesthetist. MRI protocols and sequences varied between institutions, but all the MRI examinations were acquired with dogs under general anesthesia, using high-field-strength magnets: 1.5 Tesla (Hallmarq PetVet; Siemens Magnetom Sola; Siemens SymphonyTim; Philips Medical Systems Ingenia CX). MRI sequences included at least T2-weighted (T2W) sagittal, dorsal and transverse planes. Other additional sequences acquired were: T2W DIXON (Fat and water) dorsal, T2^*^-weighted gradient echo (T2^*^) transverse, T1-weighted (T1W) pre- and post-contrast transverse and sagittal, T2W Half-Fourier Acquisition single-shot Turbo Spin Echo (HASTE) sagittal and Short-Inversion Recovery (STIR) dorsal. Contrast studies were acquired in three patients by intravenous administration of 0.2 mg/kg of Gadoteridol (Bracco, Singen, Germany). The MRI studies were reviewed by two observers independently [a certified radiologist (IC) and a ECVDI resident (DC)], followed by collective consensual evaluation using a PACS workstation DICOM viewer (OsiriX Imaging Software, 12.0MD, Berne, Switzerland). The studies were reviewed individually, followed by a consensual evaluation. Reviewers were aware of the history, patient signalment, clinical, and neurological examination findings.

The MRI study evaluation was assessed according to the following criteria: (1) the number and shape of vertebrae and the absence or presence of skeletal lesions. (2) The intervertebral discs were evaluated by size, shape, position and signal intensity of the nucleus pulposus. The signal intensity of the intervertebral discs' nucleus pulposus was defined as hypointense if abnormal and graded as mild, moderate or severe. (3) The hypaxial and epaxial muscles were assessed for volume and any signal intensity change in all available sequences. (4) The herniated disc material was described regarding margination (smoothly/well-marginated or ill-defined) and signal intensity in all sequences (homogeneous or heterogeneous, and defined as isointense, hypointense, or hyperintense compared to spinal cord). (5) The contrast enhancement pattern was defined as diffuse, rim enhancement or heterogeneous. (6) The location of the herniated disc material was attempted to be differentiated between extradural and/or intradural/extramedullary location, as follows: (A) Extradural lesion defined as a lesion which deviates and/or obliterates the epidural fat, the subarachnoid space and spinal cord (31); (B) Intradural/extramedullary lesion was defined when one or more of the following signs were found: (B.1) “Golf tee sign” defined as widening of the subarachnoid space cranial and caudal to the lesion or focal dilation/expansion of the subarachnoid space along the cranial and caudal margins of the intradural mass lesion (32); (B.2) “Y sign” defined as two hyperintense lines appearing as a “Y” representing the division of the dura and the arachnoid layers by the intradural lesion (26); (B.3) “Beak sign”: sharply marginated compressing lesion with a pointed/sharp beak-like appearance or appreciated as an increased signal intensity area surrounding the herniated disc, with a sharp beak-like appearance.

The cranial and caudal extent of the extradural or intradural lesion was measured in relation to the intervertebral disc over which the lesion was centered, using the vertebral length as a reference.

The degree of spinal cord compression caused by the intradural/extramedullary lesion was judged subjectively as mild, moderate or severe. The presence of any intramedullary lesions accompanying the compressive lesion were also noted, describing its margination, extension, signal intensity in all sequences and presence or absence of contrast enhancement.

## Results

### Signalment and clinical findings

Six dogs met the inclusion criteria but a total seven cases were evaluated as one dog had two episodes of intradural/extramedullary disc extrusion at different times and different location (case 4 and 5). Breeds included miniature Dachshund (*n* = 3), French bulldog (*n* = 1), Jack Russell terrier (*n* = 1), and cross breed (*n* = 1). There were five male (four intact, one neutered) and 1 female (neutered) and the median age was 11 years (range of 4–14 years).

The signalment, presenting neurological signs and location of intradural/extramedullary disc extrusion are included in Table 1. A history of acute non-ambulatory paraparesis and positive nociception was present in all the cases included in this study. Hyperesthesia at the level of the thoracolumbar junction was present in three cases (case 1, 2, and 7) and a cutaneous trunci cut-off was elucidated in other three cases at different levels (case 1, 2, and 6). The neurolocalization was T3-L3 except in case 1 which was T3-L6. All dogs had an MRI study performed within 48 h of the onset of neurological clinical signs.

**Table 1:** Clinical characteristics of seven dogs with confirmed thoracolumbar intradural disc extrusion.

**Case**	**Age (years)**	**Sex**	**Breed**	**Previous surgery**	**History**	**Neurolocalization**	**Outcome**	**IDH**	**IVDP**	**CUTANEOUS TRUNCI**	**NOCICEPTION**	**HYPERESTESIA**
1	14	FN	Dachshund	T12-T13^*^; C7-T1^*^; L2-L3^*^	Acute non-ambulatory paraparesis	T3-L6	Good	L3-L4	T8-T9 to L7-S1	T13	Y	Y
2	5	M	French bulldog	N	Acute non-ambulatory paraparesis	T3-L3	Euthanasia^∧^	L2-L3	L1-L2 to L5-L6	L3	Y	Y
3	11	M	Dachshund	N	Acute non-ambulatory paraparesis	T3-L3	Good	T11-T12	T9-T10 to L5-L6	N	Y	N
4	11	M	Dachshund	N	Acute non-ambulatory paraparesis	T3-L3	Good	L2-L3	T12-T13 to L4-L5	N	Y	N
5	11	M	Dachshund	L2-L3^**^	Acute ambulatory paraparesis	T3-L3	Good	L3-L4	T12-T13 to L4-L5	N	Y	N
6	9	M	Jack Russell terrier	N	Acute lameness to acute non-ambulatory paraparesis	T3-L3	Good	T11-T12	T12-T13, L2-L3, L3-L4	T13	Y	N
7	4	MN	Cross breed	N	Two week lameness to acute non-ambulatory paraparesis	T3-L3	Good	T11-T12	T10-T11	N	Y	Y

M, male; MN, male neutered; FN, female neutered; IDH, intradural/extramedullary disc extrusion; IVDP, intervertebral disc protrusion; Y, Yes; N, No.

* Hemilaminectomy (Intervertebral disc extrusion)-

** Durotomy (intradural/extramedullary disc extrusion).

∧ Euthanasia due to development of myelomalacia.

Surgical confirmation of intradural/extramedullary disc extrusion by durotomy was obtained in all the cases (7/7).

The outcome in the majority of the cases was good (6/7). This was considered as ambulatory without assistance and neurologically normal 3 months after surgery. One dog (case 2) was euthanised due to neurological deterioration 48 h post-surgery.

### MRI findings

The number and shape of the vertebrae were normal in the majority of the cases apart from case number two, where ventral aplasia of the 9th thoracic vertebra was identified (i.e., dorsal hemivertebra).

The intervertebral disc signal intensity was moderately T2W hypointense in all dogs. The hypaxial and epaxial muscles in all cases were normal in volume and in signal intensity in the available sequences.

The herniated disc material was identified at the level of L3-L4 intervertebral space (*n* = 2), L2-3 intervertebral space (*n* = 2) and T11-T12 intervertebral space (*n* = 3). Ill-defined margins of the lesion were appreciated in three cases, while the other four lesions showed well-defined margination. The signal intensity of the lesion relative to the spinal cord varied between being homogeneously hypointense in all sequences (*n* = 2), homogeneously hyperintense in T2W (*n* = 1), homogeneously hypointense in T2W (*n* = 1), heterogeneously hyperintense in T2W and T2^*^ (*n* = 3), partial signal void in T2^*^ (*n* = 1), heterogeneously in T2^*^ (*n* = 1) and isointense in T1W (*n* = 1). Three patients received contrast media (Gadobutrol 1 mmol/mL; Gadovist^®^) and all showed mild peripheral rim enhancement of the intradural/extramedullary lesion.

In five cases, the herniated material was purely intradural in location. In the remaining two patients, additional extradural material was identified in combination with intradural/extramedullary material: one had a small volume of disc material (case 1), whereas the other case had a large volume of mixed disc material and hemorrhage (case 2). The degree of spinal cord compression was severe in three cases (case 3, 5, and 6), moderate in three cases (case 1, 4, and 7), and mild in one case (case 2). Focal intramedullary lesions were observed in all cases at the level of the compressive lesion. These intramedullary lesions were ill-defined, affecting both gray and white matter, homogeneously hyperintense in T2W. In the dogs in which post-contrast and T2^*^ images were available, no intramedullary contrast enhancement or signs of intramedullary hemorrhage were identified (Table 2). These intramedullary lesions were suggestive of spinal cord contusion or edema secondary to the intradural/extramedullary disc extrusion.

**Table 2:** Summary of MRI findings.

**Case**	**ID**	**Compression**	**ED**	**Location**	**Margination**	**Homogeneous vs. heterogeneous**	**GTS**	**Y sign**	**Beak sign**	**Contrast enhancement**	**IM lesions**
1	Y	Moderate	Small	L3-L4	Poor	Heterogeneous	N	Y	N	Peripheral	Y
2	Y	Mild	Large	L2-L3	Poor	Heterogeneous	N	Y	N	Peripheral	Y
3	Y	Severe	N	T11-T12	Well	Homogeneous (hypointense)	N	Y	Y	N/A	Y
4	Y	Moderate	N	L3-L4	Poor	Heterogeneous	N	Y	Y	Peripheral	Y
5	Y	Severe	N	L2-L3	Well	Homogeneous (hyperintense)	N	Y	N	N/A	Y
6	Y	Severe	N	T11-T12	Well	Homogeneous (hypointense)	N	Y	Y	N/A	Y
7	Y	Moderate	N	T11-T12	Well	Homogeneous (hypointense) T2W; Heterogeneous T2*	N	Y	Y	N/A	Y

ID, intradural; ED, extradural; GTS, “golf tee sign”; IM, intramedullary; Y, Yes; N, No.

**Figure 1:**
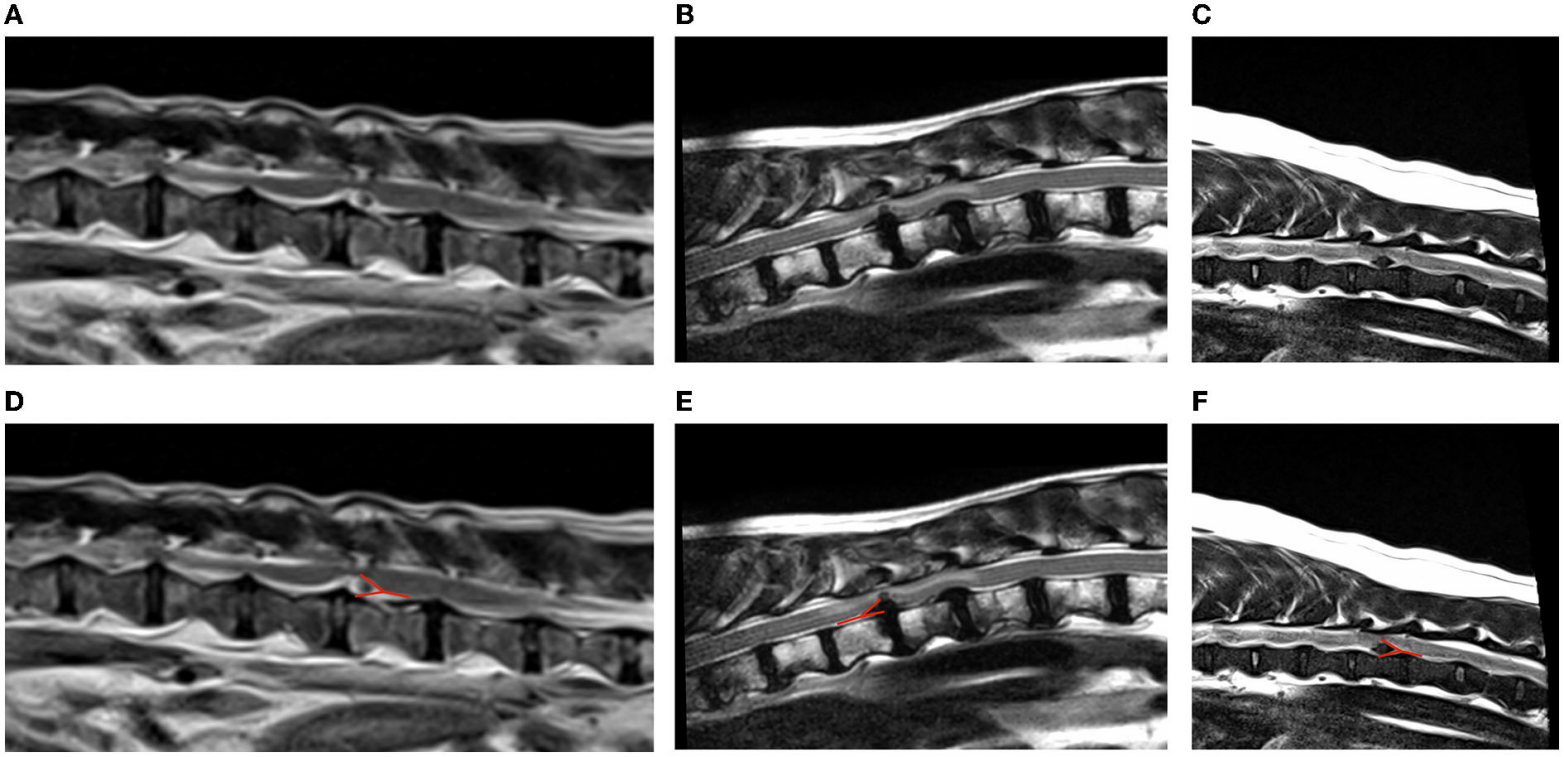
Examples of “Y sign” on T2-weighted sagittal magnetic resonance images of dogs with intradural/extramedullary disc extrusion **(A–C)**. The red line in **(D–F)** highlights the “Y sign” appearance. **(A,D)** T2W sagittal sequence showing a L3-L4 intradural/extramedullary disc extrusion with the presence of a “Y sign” (Case 4). The T2-weighted homogenous hypointense disc material is appreciated surrounded by the T2W hyperintense CSF within the subarachnoid space giving a “Y” shape. This is caused by the division of the dura and the arachnoid layers as the latter is peeled off from the dura by the disc herniation which occurs between the dura and the arachnoid. **(B,E)** T2-weighted sagittal sequence showing a T11-T12 intradural/extramedullary disc extrusion (Case 6) with the presence of a “Y sign.” This is represented by T2-weighted hyperintense CSF in the caudodorsal and caudoventral aspects of the disc material. This “Y” shape is given by the separation of the dura and the arachnoid layers by the intradural disc material. Consequently, T2-weighted hyperintense signal (cerebrospinal fluid) is appreciated at the edges of the lesion displayed as a “Y” shape. **(C,F)** T2-weighted sagittal sequence showing a T11-T12 intradural/extramedullary disc extrusion (Case 7) with the presence of a “Y sign.” The presence of disc material within the subdural space creates a “Y” shape which corresponds to the splitting of the dura matter and the arachnoid matter in two separate lines “Y sign.”

**Figure 2:**
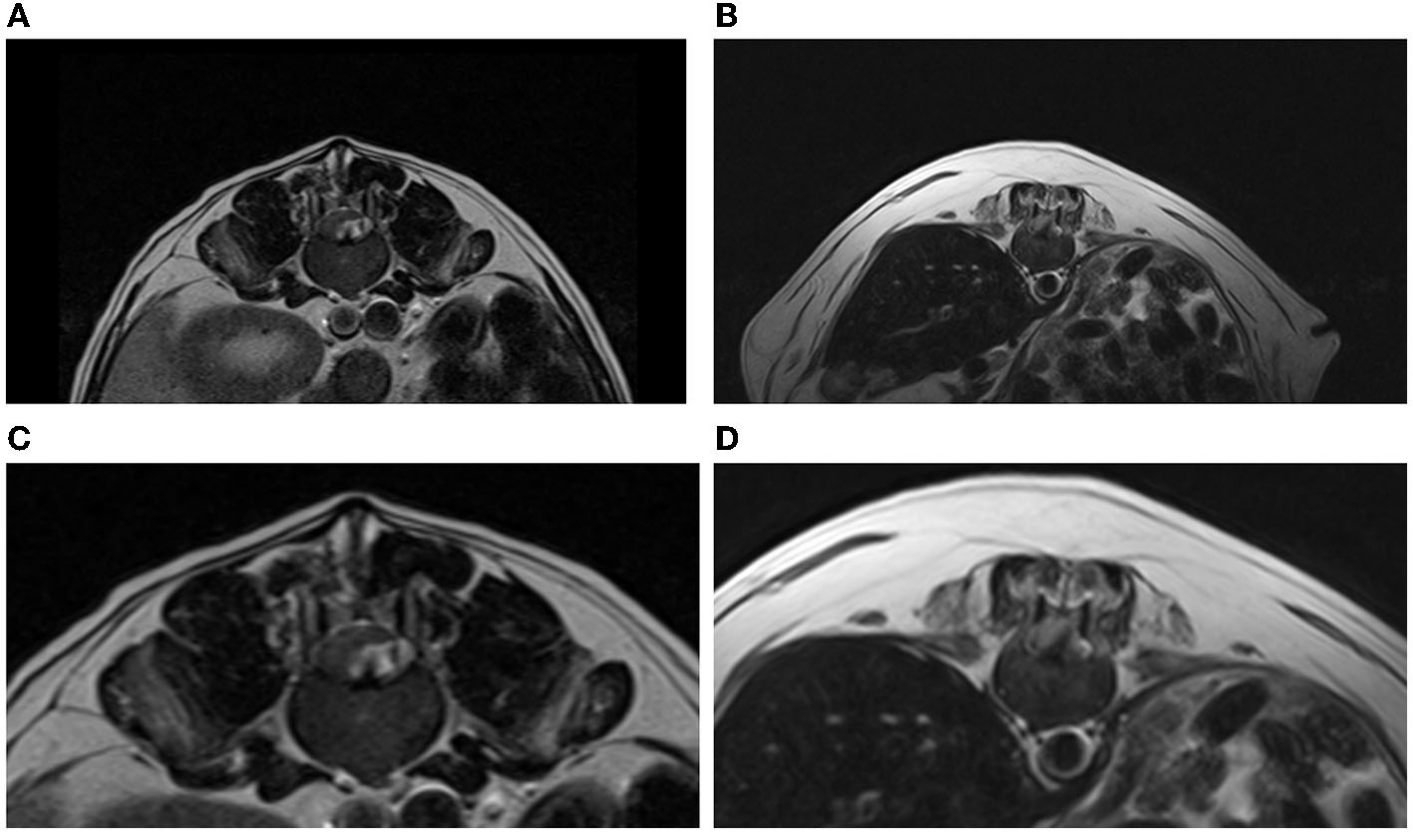
**(A,C)** T2-weighted transverse sequence showing a L3-L4 intradural/extramedullary disc extrusion (Case 4). The T2-weighted homogeneous hypointense disc material is appreciated on the left side compressing moderately the spinal cord ventrally and to the right. This is appreciated as a beak-like shaped and sharply marginated lesion (“beak sign”). The T2-weighted hyperintense signal within the subarachnoid space representing CSF is appreciated surrounding the intradural disc material. **(B,D)** T2-weighted transverse sequence displaying a T11-T12 intradural/extramedullary disc extrusion with the presence of a “beak sign” (Case 3). This is appreciated as a sharply marginated T2-weighted hypointense lesion that is compressing the spinal cord to the right side.

The “Y sign” was recognized in all the cases in the T2W sagittal plane suggesting the presence of intradural/extramedullary disc material (Figure 1). However, this was not seen in the other planes (transverse and dorsal). The “golf tee sign” was not identified in any of the dogs included. Additionally, the “beak sign” was documented in the transverse plane of more than half of the patients evaluated (4/7) (Figure 2). The MRI findings are summarized in Table 2.

## Discussion

This study describes the MRI features of surgically confirmed intradural/extramedullary disc extrusions. The “Y sign” was identified in all MRI studies included in this article, while “golf tee sign” was not appreciated in any case.

The “Y sign” has been described here for the first time in veterinary medicine, and the authors postulate that it may be an useful MRI feature for the diagnosis of intradural/extramedullary disc extrusion. This is considered of great importance with regards to preoperative diagnosis of this type of disc herniation allowing surgeons to organize a suitable surgical plan and inform on a perhaps differing prognosis.

Intradural/extramedullary disc extrusion is very rare in both human and veterinary medicine with a reported prevalence of 0.04–1.1 and 0.5%, respectively (11, 22). Even though there are few theories proposed in the human literature, the pathophysiology that causes the disc to penetrate the dura is unclear. One of them is the “acute pressure” theory that is explained as abruptly increased pressure in the intervertebral disc caused by a sudden external force on the spine. When associated with ruptured annulus fibrosus, the nucleus pulposus may break through the annulus fibrosus, dorsal longitudinal ligament and dura mater causing an intradural/extramedullary disc extrusion (22). None of the cases included in the present study had history of a traumatic event and most of the cases (4/7) were over 8-years-old. Similar to human medicine, this suggests the possibility that a chronic and degenerative process affecting the intervertebral disc is more likely to be the cause of the extrusion, rather than an acute and traumatic event. In the chronic compression theory, it is thought that chronic mechanical compression of structures such as intervertebral discs or osteophytes could make the dura mater weaker and thinner facilitating penetration of the nucleus pulposus through the dura mater to the dural sac (33). The dogs included in the present study, had multiple sites of intervertebral disc protrusion along the thoracolumbar spine which, theoretically, could have caused damage to the dura matter via chronic mechanical compression. Therefore, the authors believe that the presence of intervertebral disc protrusions could have influenced the development of dura matter thinning predisposing to intradural/extramedullary disc extrusion.

The suspicion of a chronic-on acute rather than traumatic and acute event may be also supported by the peripheral contrast enhancement surrounding the disc fragment in T1W post-contrast images, identified in the cases where these images were available (3/7). In people, this rim enhancement is believed to be caused by a reactive inflammatory response with formation of vascular granulation tissue around the chronic herniated material (30). In contrast, in acute cases, the herniated disc material has not yet been sequestered by any granulating tissue and has therefore not developed the vascularization which is the basis of the rim enhancement (20).

Adhesion formation between the ventral wall of the dura matter and the dorsal longitudinal ligament (24, 34–38) is another theory which also proposes a chronic process as a potential cause of intradural/extramedullary disc extrusions. It is thought that firm adhesions are formed between the ventral wall of the dural sac and the dorsal longitudinal ligament, which can be congenital or acquired (secondary to previous surgery or chronic inflammation occurring postoperatively) (36, 38). Therefore, in cases of dural perforation, the herniated disc material would perforate the annulus fibrosus, the dorsal longitudinal ligament, and the dura mater as if they were one structure (36, 38). Furthermore, adhesions in people occur more commonly at the L4-5 intervertebral disc space, due to increased motion and reduce stiffness which predisposes to adhesion formation (38–40). It is important to emphasize that even though the pathophysiology of the cause of the intradural-extramedullary disc extrusion may be chronic process (development of adhesions), the herniation event itself occurs acutely. This may also be supported by the presence in all cases of focal T2W hyperintense intramedullary lesions, which were suggestive of spinal cord contusion and spinal cord edema. This feature than can be explained due to the lack of protection provided by the dura matter, and thus the disc material extruding beyond it could cause contusion/damage to the cord.

In cases 4 and 5, the macroscopic appearance of the material removed from the subdural space was the same present routinely in intervertebral disc extrusion suggesting acute herniation. Unfortunately, the remaining surgical reports did not provide sufficient detail to extrapolate on how acute the disc material was.

The presence of adhesions in the dogs included in this study could not be proven intraoperatively. However, it is possible that adhesions could have been broken down by the surgeon while approaching the dura mater. The only method to confirm or rule out the presence of adhesions is by post-mortem examination, which was not possible in this study as all the dogs but one (case 2) had good outcome and were still alive at the point of writing this manuscript. Regarding a congenital cause, the fact that in this study 3 dogs were Dachshunds and one of these dogs had two intradural/extramedullary disc extrusions at different locations and different times, could suggest a congenital/embryological malformation component for adhesion development with a possible breed predisposition. However, larger number of cases are needed to prove this suspicion. In the cases included here, the location of the extrusion varied between T11-12 and L3-4. It has been proven in dogs that the removal of the dorsal longitudinal ligament causes a significant reduction in stiffness (40), and this may be similar to what is reported to happen in people at the level of L4-5 (36, 38).

The imaging diagnosis of intradural/extramedullary disc extrusions is challenging in both human and veterinary patients. There is controversy in people regarding which modality (CT myelography vs. MRI) is more reliable to identify intradural disc material (21, 41). To date, the only large study evaluating intradural/extramedullary disc extrusion in dogs has proposed that CT myelography may be more valuable demonstrating this type of disc extrusion than low-field MRI (11). This was based on the observation of a “golf tee sign” and a filling defect on CT myelography. There were, however, no clear low-field MRI findings suggesting the presence of disc material within the dural space. Consequently, all the cases in that study were preoperatively misdiagnosed on MRI as extradural intervertebral disc extrusion. The reason for this may be the poorer resolution of low-field MRI in comparison with high-field MRI, in addition to the thicker slices and larger slice gaps used with low-field MRI, which may prevent the visualization of subtle changes within the meninges.

The fact that the “Y sign” was observed in all dogs in this study may suggest that this could be a characteristic MRI feature of intradural/extramedullary disc extrusions, supported by the theory of how the disc material may penetrate the dura. As explained before, there appears to be a chronic process predisposing to dural penetration by an acute disc extrusion. When disc material exists between the dura and the arachnoid, the dura and arachnoid line is divided into two lines due to the presence of material within the subdural space giving an appearance of a “Y” (42). In human medicine this MRI feature has been reported as an indicator of intradural/extramedullary disc extrusion as the arachnoid is peeled from the dura by the disc herniation (26, 42, 43, 47), and this is in accordance with the findings in the study presented here. Further studies with a larger number of patients would be beneficial to confirm this MRI feature of “Y sign.” In addition, it would be of interest to investigate if the “Y sign” may be also seen in other intradural/extramedullary pathologies, such as neoplasia, and to compare the presence of the “Y sign” vs. “golf tee sign” on MRI.

The focal expansion of the subarachnoid space along the cranial and/or caudal margins of the intradural/extramedullary component of a lesion can form a “golf tee sign” on MRI similar to what observed on myelographic images, particularly on sagittal or dorsal plane images (depending on the location of the lesion relative to the spinal cord) (32). A “golf tee sign” appears when the contrast column diverges to encompass the material within the subarachnoid space and it has been proposed as a hallmark of all intradural/extramedullary lesions including herniations and tumors (meningioma or hemangiosarcoma) (11, 41, 44, 45). Differentiation between these two processes has been discussed in humans because similar MRI findings have been reported (46). The gradual growth pattern of neoplastic processes could explain the progressive expansion of the subarachnoid space cranially and caudally to accommodate the mass giving rise to the “golf tee sign.” In contrast, an intradural/extramedullary disc extrusion occurs acutely as the result of dura mater tear/rupture. Consequently, the disc material migrates rapidly through the subdural space and thus the subarachnoid space does not have time to accommodate and adopt the shape of the material. This may be of the reasons why we did not identify “golf tee sign” in this study. Another reason could be that no high-resolution and thin slice sequences without slap gap were included in any of the multiple MRI protocols in the cited study. A further explanation for the lack of “golf tee sign” in the present study may be due to the higher viscosity and thickness of non-ionic contrast medium in comparison to cerebrospinal fluid. The former cannot enter into smaller spaces (dura and arachnoid) and thus gets trapped in the subarachnoid space just before the meningeal splitting giving the appearance of a golf tee.

The clinical presentation of all the cases presented in this study include a non-ambulatory paraparesis clinical status. Despite the low number of patients included in this study, the fact that all dogs were non-ambulatory may be a characteristic of intradural/extramedullary disc extrusions.

The limitations of this study are related to its retrospective and multicentric nature resulting in a small number of cases and the lack of high-resolution MRI sequences in all the cases. However, the authors believe that the dataset provided was representative of what a radiologist may find in routine clinical work.

In conclusion, the authors propose “Y sign” as a reliable high-field MRI feature for the diagnosis of intradural/extramedullary disc extrusion. Even though it is very uncommon in veterinary medicine, the presence of “Y sign” in high-field MRI may aid the radiologist and neurologist to diagnose intradural/extramedullary disc extrusion preoperatively and be aware of the intraoperatively findings.

## Data availability statement

The raw data supporting the conclusions of this article will be made available by the authors, without undue reservation.

## Ethics statement

The animal study was reviewed and approved by the Ethical Review Committee of the University of Nottingham School of Veterinary Medicine and Science. Written informed consent for participation was not obtained from the owners because the animals were having an MRI study regardless.

## Author contributions

IC conceived of the presented idea. DC and IC performed the image evaluation. DC wrote the manuscript. All authors contributed cases and contributed to the final manuscript. All authors contributed to the article and approved the submitted version.

## Funding

Linnaeus Veterinary Limited supported the costs of the Open Access Publication Charges.

## Conflict of interest

The authors declare that this study received funding from Linnaeus Veterinary Limited. The funder was not involved in the study design, collection, analysis, interpretation of data, the writing of this article, or the decision to submit it for publication.

## Publisher's note

All claims expressed in this article are solely those of the authors and do not necessarily represent those of their affiliated organizations, or those of the publisher, the editors and the reviewers. Any product that may be evaluated in this article, or claim that may be made by its manufacturer, is not guaranteed or endorsed by the publisher.
